# Seed Dormancy and Germination Responses of *Salicornia brachiata*: Towards Sustainable Cultivation and Conservation in Saline Habitats

**DOI:** 10.3390/plants14131893

**Published:** 2025-06-20

**Authors:** Sandani M. Gunasekara, Chamara L. Mendis, Dinum Perera, Malaka M. Wijayasinghe

**Affiliations:** 1 Department of Bioprocess Technology, Faculty of Technology, Rajarata University of Sri Lanka, Mihintale 50300, Sri Lanka; sandanigunasekara4@gmail.com (S.M.G.); dchamara1997@gmail.com (C.L.M.); 2 Faculty of Applied Sciences, Rajarata University of Sri Lanka, Mihintale 50300, Sri Lanka; 3 Academic and Student Administration, Oxford Brookes University, Oxford OX3 0BP, UK

**Keywords:** physiological dormancy, gibberellic acid, halophyte, saline agriculture, perianth, seed morphology, germination temperature, geographic location

## Abstract

*Salicornia brachiata* Roxb., an economically and ecologically significant halophytic species native to Sri Lanka, produces dimorphic seeds. Despite their importance for commercial cultivation and conservation, germination studies of these dimorphic seeds remain limited. This study investigated the effects of temperature (25 °C, 30 °C, 35 °C), gibberellic acid (GA_3_) treatment, geographic location of seed source (Jaffna vs. Puttalam coastal regions), seed type (central vs. lateral), and perianth presence/absence on germination under controlled conditions. Our results show that temperature, GA_3_, and geographic location of the seed source significantly influenced seed germination. This study presents the first documented evidence of physiological dormancy (PD) in *S. brachiata* seeds, with successful dormancy breaking achieved using GA_3_ treatment at 25 °C. Although perianth and seed type alone had no significant direct effects on germination, they were involved in multiple significant interactions—two-, three-, and four-way—with other factors. These findings highlight the multifactorial regulation of *S. brachiata* seed germination, suggesting that tailored propagation strategies, incorporating environmental and physiological variables, can optimize germination. These findings offer practical solutions for enhancing germination in saline agriculture and habitat restoration efforts of *S. brachiata*.

## 1. Introduction

*Salicornia brachiata* Roxb. is a halophytic herb native to Sri Lanka and commonly found across the Indian subcontinent to Myanmar. This species holds significant promise for applications in functional foods, nutraceuticals, and pharmaceutical formulations due to its bioactive properties and its biomass has potential as a feedstock for biofuel generation [1,2,3,4,5]. In addition to its commercial value, *S. brachiata* plays a crucial role in environmental conservation and land rehabilitation. The species demonstrates high capacity for phytoremediation of saline soils, making it an important tool for restoring degraded coastal ecosystems [6]. This dual significance—as both an economic resource and an ecological engineer—positions *S. brachiata* as a species of particular interest for sustainable development initiatives in saline-affected regions.

To meet the growing demand for the above-mentioned applications, the commercial cultivation of *S. brachiata* is essential. This would not only ensure a stable supply but also reduce reliance on wild populations and help conserve natural habitats [7]. Introducing the commercial cultivation of *S. brachiata* in saline and degraded areas offers a dual benefit: providing novel, health-promoting food sources and addressing critical environmental concerns related to biodiversity conservation [8].

However, to enable sustainable cultivation, it is crucial to develop comprehensive knowledge of seed dormancy and germination, as these traits directly influence plant establishment, stress tolerance, biomass production, and yield potential [9]. Yet, research on seed dormancy and germination behavior in *S. brachiata* remains limited, particularly regarding the roles of seed traits such as dormancy, dimorphism, structural features and abiotic factors such as temperature and chemical regulators. Filling these knowledge gaps is vital to support the sustainable cultivation, conservation, and commercial viability of this species.

Seed dormancy—the failure of viable seeds to germinate under favorable environmental conditions—poses a major challenge to achieving synchronized germination and uniform seedling development, which are critical for successful commercial cultivation [10]. However, dormancy can also be advantageous for plant restoration efforts by enhancing seed survival through the formation of a soil seed bank [10,11]. Thus, understanding the mechanisms of dormancy in *S. brachiata* is crucial.

Various physical, chemical, and physiological methods have been employed to break dormancy in *Salicornia* species across different regions. Among these, cold stratification has proven effective in species such as *S. europaea* and *S. patula,* which are native to temperate and Mediterranean climates, respectively [12,13]. Additionally, the exogenous application of gibberellic acid (GA_3_) has successfully enhanced germination and seedling growth in several *Salicornia* species, including *S. europaea*, *S. pacifica*, and *S. rubra.* [12,13,14]. However, identification of seed dormancy class of *S. brachiata* has not been studied yet, considering the dimorphic nature of the seeds [11].

In *Salicornia* species, seed dimorphism—the production of two distinct types of seeds by a single plant—is an evolutionary bet-hedging strategy that enhances a species’ ability to survive under unpredictable or fluctuating environmental conditions [15,16,17]. In *Salicornia* species, seed dimorphism is evident; large seeds develop from central flowers, while smaller seeds originate from lateral flowers within the cymule inflorescence [11,18]. In general, dimorphic seeds differ significantly in dormancy and germination responses [11,18]. This is achieved by equipping seeds with differing morphological and physiological traits, such as variations in size, dispersal ability, dormancy requirements, germination timing, and seed bank dynamics [19,20,21]. Germination studies on *S. europaea*, *S. ramosissima*, and *S. patula* [22,23,24] reported that lateral seeds exhibit greater dormancy and are more sensitive to salinity stress, often requiring dormancy-breaking treatments such as cold stratification or light exposure to germinate, while central seeds tend to germinate more readily under favorable conditions.

In general, seed structural features such as the funiculus, hilum, micropyle, and perianth play key roles in seed development, germination, and dispersal [25]. In *Salicornia*, most dried seeds are enclosed within an intact perianth, which is composed of the sepals and petals of the flower and may act as a physical barrier or provide chemical inhibition during the germination process [26]. Despite the use of optimum environmental conditions for germination, *S. herbacea* showed a low germination percentage (28%), which dramatically increased to 95% following the removal of the funiculus—the seed stalk that attaches the ovule to the placenta. However, removal of the perianth had no significant effect on germination [26].

Previous germination studies in *Salicornia* species [13,14,23] have reported the complex interactions among seed traits and abiotic factors. A deeper understanding of these interactions is fundamental to optimizing both the commercial cultivation and conservation of *S. brachiata*. Although a previous study has explored aspects of seed germination under salinity and osmotic stress in this species [27], comprehensive research on the combined effects of seed dimorphism, perianth presence, temperature, and GA_3_ application remains scarce.

Given the critical role of *S. brachiata* in advancing sustainable agriculture and conservation initiatives, investigating its seed dormancy and germination responses is essential. This knowledge will contribute to the development of innovative cultivation strategies that enhance germination efficiency, support biodiversity conservation, and enable the sustainable utilization of saline habitats. Therefore, the present study aims to assess the interactive effects of temperature and chemical priming on seed dormancy and germination in *S. brachiata*, with particular emphasis on seed morph (central vs. lateral), perianth presence, and geographic location of the seed source.

## 2. Results

### 2.1. Individual Effects of Temperature, Perianth Presence, Seed Type, Chemical Treatment and Geographic Location of the Seed Source on the Seed Germination Percentage of S. brachiata

A significant individual effect on the germination percentage (GP) was observed only for temperature, GA_3_ and geographic location. Seed type and perianth presence did not show a significant effect on GP (Table 1).

**Figure 1:**
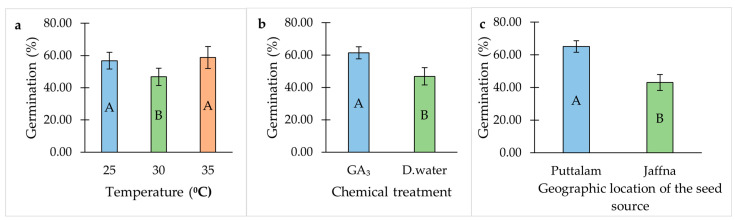
Individual effect of temperature, chemical treatment and geographic location of the seed source on the germination percentage of *Salicornia brachiata* seeds. (**a**) Effect of temperature on the germination percentage of *Salicornia brachiata* seeds. (**b**) Effect of chemical treatment on the germination percentage of *Salicornia brachiata* seeds. (**c**) Effect of geographic location of the seed source on the germination percentage of *Salicornia brachiata* seeds. Bars represent mean germination percentages ± standard error. Bars with different uppercase letters within each graph are significantly different (*p* < 0.05) according to Tukey’s test.

Seeds exhibited the highest GP at 25 °C and 35 °C, whereas the lowest germination was recorded at 30 °C (Table 1, Figure 1a). The application of gibberellic acid (GA_3_) significantly improved GP compared to the distilled water treatment (Table 1, Figure 1b). Furthermore, seeds collected from Puttalam district showed the highest GP compared to those from Jaffna district (Table 1, Figure 1c).

### 2.2. Interaction Effects of Temperature, Perianth Presence, Seed Type, Chemical Treatment and Geographic Location of the Seed Source on Seed Germination Percentage of S. brachiata

The analysis shows significant multi-factor interactions affecting seed GP, including two-way, three-way, and four-way interactions (Table 1, Supplementary Material Figure S1, Table S1).

**Figure 2:**
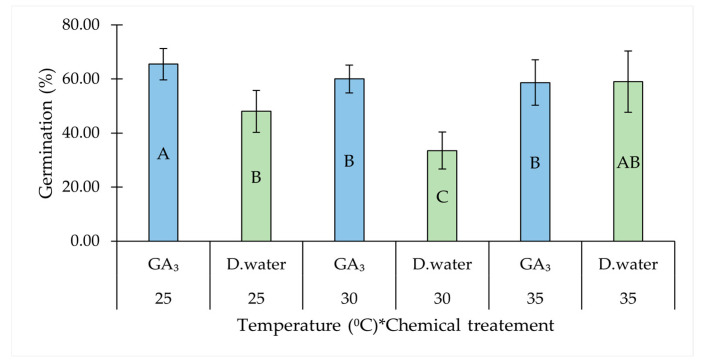
Interaction effect of temperature and chemical treatment (GA_3_) on the germination percentage of *Salicornia brachiata* seeds. Bars represent mean germination percentages ± standard error. Values at each factor combinations having different uppercase letters are significantly different (*p* < 0.05) according to Tukey’s test. Mean germination percentages were compared between GA_3_ and water treatments at each temperature, as well as across all combinations of chemical treatments and temperatures. The asterisk (*) denotes an interaction effect between the factors.

Among the two-way interactions, temperature and chemical treatment showed a notable interactive effect. GA_3_-treated seeds showed the highest GP at 25 °C, significantly improving germination compared to the control (with distilled water) at both 25 °C and 30 °C. However, this was not evident at 35 °C between GA_3_-treated and untreated seeds (Table 1, Figure 2).

**Figure 3:**
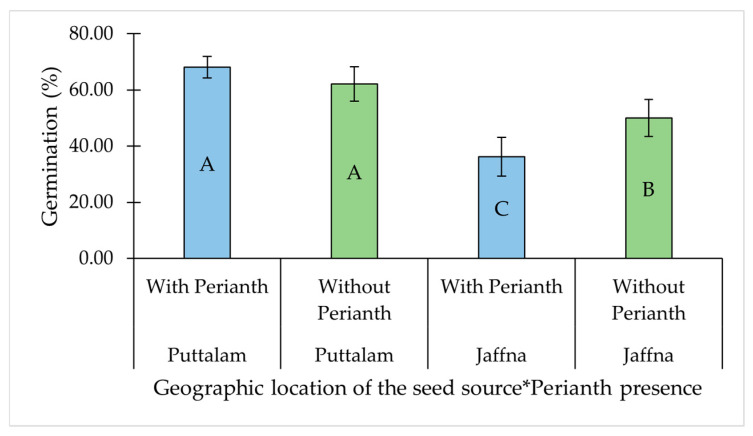
Interaction effect of geographic location of the seed source and perianth presence on the germination percentage of *Salicornia brachiata* seeds. Bars represent mean germination percentages ± standard error. Bars at each factor combination having different uppercase letters are significantly different (*p* < 0.05) according to Tukey’s test. Mean germination percentages were compared between perianth presence and absence at each geographic location, as well as across all combinations of geographic location and perianth status. The asterisk (*) denotes an interaction effect between the factors.

The other significant two-way interaction was observed between the perianth presence and geographic location of the seed source (Table 1). While perianth presence had no significant effect on GP in seed collected from Puttalam, its removal significantly improved germination in seeds collected from Jaffna (Table 1, Figure 3).

Observed three-way interactions in our study were temperature, perianth presence, and seed type (T × P × S); temperature, perianth presence, and chemical treatment (T × P × Chem); perianth presence, seed type, and chemical treatment (P × S × Chem); and perianth presence, chemical treatment, and geographic location of the seed source (P × Chem × GL). These interactions significantly influenced GP, with variations observed depending on the specific combination of factors (Table 1).

Significant four-way interactions were observed, which influenced GP in distinct ways depending on the combinations of factors. These included temperature, perianth presence, seed type, and geographic location of the seed source (T × P × S × GL); perianth presence, seed type, chemical treatment, and geographic location of the seed source (P × S × Chem × GL); and temperature, perianth presence, chemical treatment, and geographic location (T × P × Chem × GL) (Table 1).

## 3. Discussion

Our results demonstrate that temperature significantly influenced the germination percentage (GP) of *S. brachiata* seeds. Interestingly, seeds showed significantly higher GPs at both 25 °C and 35 °C, with a noticeable decline at 30 °C. This non-linear pattern parallels findings in other *Salicornia* species, including *Salicornia rubra* (Nels.), where optimal germination occurred at a temperature regime of 25 °C at night and 35 °C during the day, while lower temperatures (5–15 °C) significantly inhibited seed germination [28]. Similarly, *Salicornia europaea* L. exhibited non-linear radicle emergence responses to temperature gradients ranging from 10 °C to 30 °C [12]. The same nonlinear response was observed for some other *Salicornia* spp. such as *S. herbacea* [29], *S. patula* [30] and *S. pacifica* [14]. This germination behavior may reflect an adaptive strategy to fluctuating thermal conditions in the species’ natural habitat [30], where seeds likely encounter alternating periods of moderate and high temperatures. Such temperature-dependent germination plasticity could enhance establishment success across variable microclimates within coastal ecosystems [30].

The present study also revealed that GA_3_ treatment significantly improved germination percentages of *S. brachiata* seeds compared to untreated seeds, indicating the presence of physiological dormancy (PD) in a portion of the seed population, according to the seed dormancy classification system [11]. Even though PD has been previously documented in other *Salicornia* spp. [11] to our knowledge, this study represents the first report assigning dormancy class in *S. brachiata*. GA_3_ treatment also improved germination of *S. rubra* under both low- and high-salinity conditions [13]. In addition, exogenous GA_3_ applications were found to enhance germination of *S. europaea* seeds under saline conditions [12]. Identification of seed dormancy type of this species provides critical information for seed banking ex situ and habitat restoration efforts. For instance, known dormancy-breaking treatments of these species enhance the accuracy of seed viability assessments, as normal seedling development following radicle emergence serves as a more reliable viability indicator than tetrazolium staining alone. This knowledge enables improved seed storage protocols, ensuring long-term viability in ex situ conservation efforts.

Further, our study revealed a significant temperature × chemical treatment interaction, suggesting that the effectiveness of GA_3_ in breaking seed dormancy is temperature dependent. The combination of optimal germination temperature (25 °C) with GA_3_ treatment improved germination, providing implications for cultivation practices. Such optimized protocols would not only increase operational efficiency but also reduce resource inputs during the critical early growth phase. These practical applications may be particularly valuable for commercial propagation and restoration initiatives where reliable seedling production is essential. For example, the application of GA_3_ can be integrated into seed pretreatment protocols with optimal temperature to ensure uniform and rapid seedling establishment.

A previous study has reported that the interaction between incubation temperature and GA_3_ significantly enhanced seed germination of another halophyte, *Chenopodium album,* and two adaptive weed species, *Vicia sativa* and *Physalis minima.* Maximum germination percentages were observed for *Vicia sativa* (79% at 20 °C), *Chenopodium album* (69.8% at 15 °C), and *Physalis minima* (62% at 20 °C), highlighting the crucial role of temperature and GA_3_ in promoting seed germination [31]. Similarly, in *Capparis spinosa*, a shrub equipped with both halophytic and xerophytic traits, the interaction between GA_3_ concentration and temperature significantly influenced germination, where moderate GA_3_ levels enhanced germination at laboratory temperatures (35.7 °C), but higher concentrations under greenhouse conditions (43 °C) led to a substantial decline in germination percentage [32]. These studies further support the notion that temperature and GA_3_ interact in influencing seed germination, and it highly depends on the plant species, as observed in the present study. This emphasizes the importance of considering these factors in germination studies.

Additionally, the geographic location of the seed source had a significant impact on germination of *S. brachiata*. Seeds collected from Puttalam showed higher germination percentages compared to those from Jaffna, showing interpopulation dormancy variation of this species. This variation may be attributed to genetic diversity between populations or maternal effects. The environmental conditions experienced by the mother plants (e.g., soil salinity, moisture availability, nutrient levels, or climatic stress) could influence the physiological and biochemical traits of the seeds, leading to differences in dormancy or germination responses of the progeny [33,34,35,36,37]. In the present study, seeds from Puttalam had higher moisture content compared to those from Jaffna, which may have contributed to their higher germinability. Jaffna, characterized by higher soil salinity and lower rainfall, presents more stressful maternal environments, which may influence seed development through physiological adaptations associated with dormancy.

A study on *S. ramosissima* revealed significant differences in growth parameters, such as stem elongation, between the two populations, likely due to variations in salinity between the two sampling sites and/or genetic adaptations to the specific environmental conditions at each location [38]. Such population-specific responses emphasize the importance of considering seed sources in the designing restoration or cultivation programs.

The observed variation in germination performance between seeds from Puttalam and Jaffna suggests that selective breeding or the use of high-performing seed sources could enhance the germinability of *S. brachiata* in commercial settings. For example, seeds from the Puttalam population, which showed higher germination percentages, possess desirable traits for commercial cultivation compared to those from the Jaffna population. Leveraging such population-specific traits can support the development of improved *S. brachiata* varieties tailored to regional conditions, thereby increasing the economic viability of saline agriculture. These findings also highlight the importance of considering local adaptation and genetic diversity when selecting seed sources for conservation programs. Conservation efforts should prioritize the preservation of locally adapted populations to maintain the genetic resilience of the species.

The interaction between perianth presence and geographic location of the seeds highlights how local microhabitat conditions can significantly modulate seed germination responses, possibly due to morphological adaptations in seed characteristics. Although no significant interaction was detected between perianth presence and geographic location in the Puttalam district, a significant interaction was observed in the Jaffna district, where seeds exhibited enhanced germination following perianth removal. This finding suggests that geographic factors can regulate seed germination by influencing morphological characteristics, underlining the need to consider multiple environmental and biological factors when studying seed germination behavior.

These observations align with studies on other halophytes, such as *Abronia umbellata*, in which seed appendages (particularly wing size) have been found to increase in populations at the outermost boundaries of the species’ natural distribution, where environmental conditions are typically more variable and stressful. This suggests that seed morphological traits adapt to geographic location, with larger wings likely improving dispersal in these challenging areas [39]. Additionally, the variation in awn length observed among 20 populations of Siberian wildrye (*Elymus sibiricus* L.) supports the idea that environmental factors influence seed traits, affecting both seed yield and dispersal [40]. These findings collectively suggest that geographic factors play a critical role in shaping seed characteristics and germination responses, which should be considered when developing strategies for propagation and commercialization of species such as *S. brachiata*.

The complex interactions observed among temperature, chemical treatments, and seed characteristics underscore the need for a holistic approach to both conservation and commercial cultivation of *S. brachiata*. This complexity highlights the complicated balance required for successful germination, which may be critical for survival in the natural habitat of *S. brachiata*, where environmental conditions are highly variable. Such interactions may also reflect the species’ evolutionary adaptation to unpredictable saline environments [41,42,43]. These findings also suggest that, in commercial cultivation, farmers should adapt germination protocols to local temperatures and focus on post-harvest processing to optimize germination rates. By integrating these evidence-based practices, stakeholders can enhance both the conservation and commercial scalability of *S. brachiata*, promoting sustainable agricultural systems in saline-affected regions.

In this study, radicle protrusion of ≥2 mm was used as the germination criterion. However, the subsequent development of these germinated seeds into healthy seedlings remains uncertain, potentially leading to an overestimation of viable seedling percentages [44]. Further research is needed to evaluate temperature effects on post-germination viability and seedling establishment, particularly to support the large-scale commercial cultivation and ecological restoration applications of this species.

## 4. Materials and Methods

### 4.1. Seed Collection

**Figure 4:**
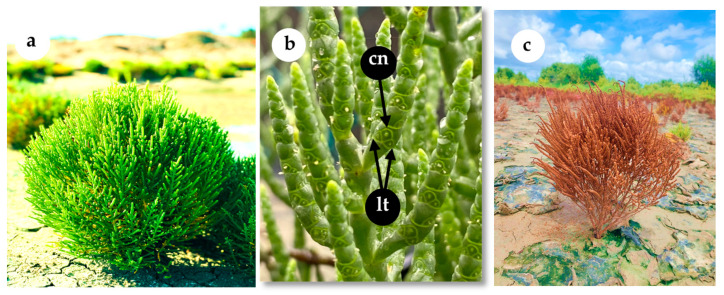
Various growth stages of *Salicornia brachiata* growing in their natural habitat (Puttalam, Sri Lanka). (**a**) Vegetative stage; (**b**) reproductive stage, showing the central flower (cn) and lateral flowers (lt); (**c**) senescence stage.

Plants at the senescence stage (Figure 4c) bearing dispersal-ready seeds were manually uprooted from two geographic locations: Karamba, Puttalam (7°58′28.0″ N, 79°48′42.5″ E) and Karainagar, Jaffna (9°44′28.5″ N, 79°53′36.9″ E) in Sri Lanka during late October 2024. Seed collection was conducted as a bulk collection, where seeds from multiple plants were pooled together without tracking individual maternal lines. Collected plants (at least 10 plants from each site) were immediately placed in labeled polythene bags, brought to Department of Bioprocess Technology laboratory, Rajarata University of Sri Lanka within a day, air-dried at room temperature (~30 °C) for 7 days, then refrigerated at 4 °C for two months before germination experiments. Seed maturity was defined based on the visual assessment of senescence stage, characterized by yellowing and drying of aboveground tissues, along with the ease of seed detachment upon gentle touch. Seed moisture content was measured prior to storage, averaging 25.7 ± 2.3% for Puttalam seeds and 13.5 ± 1.0% for Jaffna seeds. Necessary approvals were granted by the relevant authorities for sampling. Although both geographical locations fall under the tropical savanna climate, the primary difference is that soils in the Jaffna District are significantly more saline than any other places in Sri Lanka including Puttalam, due to lower rainfall, poor drainage, and the presence of a limestone bedrock [45].

### 4.2. Germination Experiment

**Figure 5:**
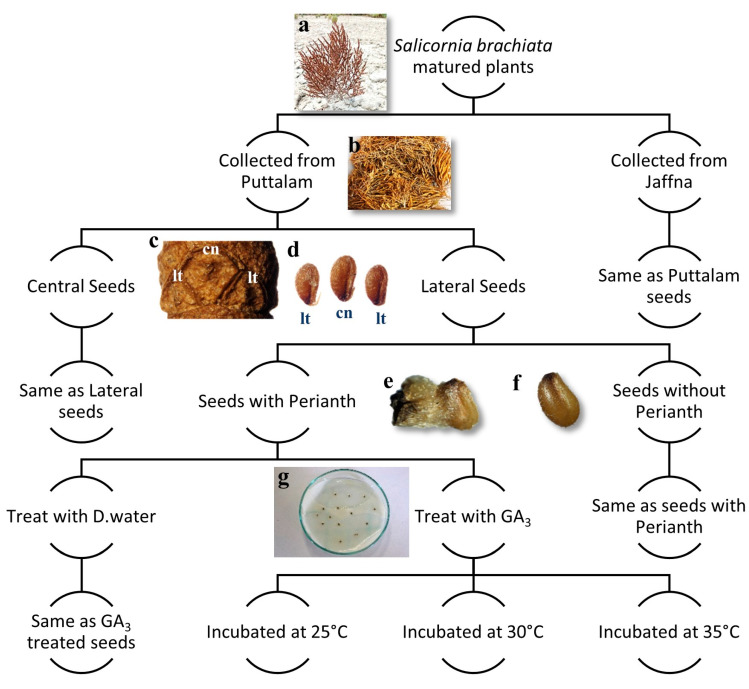
Schematic representation of the methodological workflow of seed germination experiment: (**a**) Matured and dried *Salicornia brachiata* plant in the seed senescence stage in their natural habitat; (**b**) collected shoot samples for seed extraction; (**c**) seed bearing dry cladode (stem piece) (cn) central seeds and (lt) lateral seeds; (**d**) central and lateral seeds detached from plant (perianth is also removed); (**e**) seed with the perianth segment attached; (**f**) seed without the perianth segment; (**g**) a prepared Petri dish after seed treatment.

Seeds from two locations were used separately in these experiments. Initially, the seeds were separated based on their dimorphic nature into two categories: central seeds and lateral seeds. From each category, a large subsample of seeds was further divided into two groups: seeds with intact perianths and seeds with removed perianths. This process resulted in four distinct seed samples: (1) central seeds with perianths, (2) central seeds without perianths, (3) lateral seeds with perianths, and (4) lateral seeds without perianths (Figure 5).

Prior to the experiments, all seed samples were surface sterilized by immersing them in a 2% (*v*/*v*) commercial bleach (Clorox, Oakland, CA, USA) (5.25% NaOCl) solution for 20 min with gentle agitation. After sterilization, the seeds were rinsed three times with distilled water [27,46] and air dried for 1 h at room temperature before germination experiments.

Germination tests were conducted with three replicates of 25 seeds for each of the four seed types mentioned above. The tests were performed using 9 cm Petri dishes lined with one layer of Whatman No. 1 filter paper (Cytiva, Little Chalfont, Buckinghamshire, UK). Two sets of Petri dishes were prepared; one set was moistened with GA_3_ (Duchefa Biochemie, Haarlem, The Netherlands) solution (250 ppm), and the other set was moistened with autoclaved distilled water, serving as the control treatment.

Both sets of Petri dishes were incubated under a 12-h photo period at three different temperatures: 25 °C, 30 °C, and 35 °C. Germinated seeds were recorded every three days until no further germination was observed (up to 30 days). A seed was considered germinated when the protruding radicle was ≥2 mm long. A schematic overview of the methodological workflow is presented in Figure 5.

### 4.3. Data Analysis

The germination percentage was calculated using the following formula.

GP (%) = (Number of seeds germinated/Total number of viable seeds) × 100

GP data were transformed (arcsine) before statistical analysis. Data were tested for normality and then analyzed by ANOVA. Differences between means were compared using the Least Squares Means values from Tukey’s grouping. All statistical analyses were performed using SAS software, Version 9.4 (M7) of the SAS System for UNIX (©2025 SAS Institute Inc., Cary, NC, USA). The analyses were conducted via SAS OnDemand for Academics.

## 5. Conclusions

This study provides critical information on germination ecology of *S. brachiata,* demonstrating that the seed germination is significantly influenced by the temperature, gibberellic acid (GA_3_), and geographic location of the seed source. Here, we provide the first documented evidence of physiological dormancy in *S. brachiata* seeds, showing that this dormancy can be effectively broken through GA_3_ application at 25 °C. Notably, seeds sourced from Puttalam exhibited higher germination success compared to those from Jaffna, suggesting potential local adaptations to differing environmental conditions. The complex interactions observed among factors considered in this study highlight the sophisticated germination regulation in this halophyte species and importance of developing tailored protocols for both conservation initiatives and commercial cultivation practices. Our findings offer practical insights into improving germination and promoting the use of *S. brachiata* in saline agriculture and habitat restoration. Further, it provides a scientific foundation for future studies on seed ecology and germination optimization in this economically and ecologically important halophyte.

## Figures and Tables

**Table 1:** Effects of temperature (T), perianth presence (P), seed type (S), chemical treatment (Chem), geographic location of the seed source (GL), and their interactions on germination percentage of *Salicornia brachiata* seeds according to general linear model followed by Tukey’s test. Sources with * are significantly different.

Source of Variation	DF	Type III SS	Mean Squares	F Value	*p* Value
T *	2	1.5059	0.7530	12.26	<0.0001
P	1	0.0880	0.0880	1.43	0.2341
S	1	0.0272	0.0272	0.44	0.5071
Chem *	1	1.6943	1.6943	27.60	<0.0001
GL *	1	3.3003	3.3003	53.75	<0.0001
T × P	2	0.0096	0.0048	0.08	0.9248
T × S	2	0.0690	0.0345	0.56	0.5722
T × Chem *	2	1.0119	0.5060	8.24	0.0005
T × GL	2	0.3023	0.1512	2.46	0.0906
P × S	1	0.1122	0.1122	1.83	0.1796
P × Chem	1	0.0702	0.0702	1.14	0.2875
P × GL *	1	0.4444	0.4444	7.24	0.0084
S × Chem	1	0.0235	0.0235	0.38	0.5375
S × GL	1	0.0005	0.0005	0.01	0.9305
Chem × GL	1	0.0061	0.0061	0.10	0.7526
T × P × S *	2	0.6855	0.3428	5.58	0.0051
T × P × Chem *	2	1.4700	0.7350	11.97	<0.0001
T × P × GL	2	0.2176	0.1088	1.77	0.1755
T × S × Chem	2	0.3686	0.1843	3.00	0.0544
T × S × GL	2	0.3173	0.1587	2.58	0.0807
T × Chem × GL	2	0.0778	0.0389	0.63	0.5329
P × S × Chem *	1	0.2704	0.2704	4.40	0.0385
P × S × GL	1	0.0015	0.0015	0.02	0.8774
P × Chem × GL *	1	0.9120	0.9120	14.85	0.0002
S × Chem × GL	1	0.0121	0.0121	0.20	0.6581
T × P × S × Chem	2	0.2259	0.1130	1.84	0.1644
T × P × S × GL *	2	0.5011	0.2505	4.08	0.0199
P × S × Chem × GL *	1	0.6944	0.6944	11.31	0.0011
T × P × Chem × GL *	2	1.0310	0.5155	8.40	0.0004
T × S × Chem × GL	2	0.3468	0.1734	2.82	0.0643
T × P × S × Chem × GL	2	0.0721	0.0360	0.59	0.5581

DF: Degree of freedom, Type III SS: Type III sums of square.

## Data Availability

The original contributions presented in this study are included in the article. Further inquiries can be directed to the corresponding authors.

## Supplementary Materials

The following supporting information can be downloaded at: https://www.mdpi.com/article/10.3390/plants14131893/s1, Figure S1: Forest plot of parameter estimates (±95% confidence intervals) from a full factorial ANOVA model (PROC GLM) analysing seed germination percentage (gp) of *Salicornia brachiata* seeds. The model includes five categorical factors: temperature (t), perianth presence (p), seed type (s), chemical treatment (chem), and geographic location of the seed source (GL), including all main effects and their interactions (up to five-way). The *y*-axis lists the parameter names (main effects and interactions), while the *x*-axis shows the estimated effect sizes with 95% confidence intervals calculated as estimate ±1.96 × standard error. The vertical red line at zero represents no effect; estimates to the right indicate positive effects on germination, while those to the left indicate negative effects. Table S1: Least square means (LS-means) and 95% confidence intervals for the four-way interactions among temperature (t), perianth presence (p), seed type (s), chemical treatment (chem), and geographic location of the seed source (GL) on seed germination percentage of *Salicornia brachiata* seeds. The table includes LS-means for the four-way interactions among: temperature × perianth presence × seed type × geographic location of the seed source; temperature × perianth presence × chemical treatment × geographic location of the seed source; and perianth presence × seed type × chemical treatment × geographic location of the seed source. All means were obtained according to a general linear model followed by Tukey’s test.
