# Supplementary material for: Seed Dormancy and Germination Responses of Salicornia brachiata: Towards Sustainable Cultivation and Conservation in Saline Habitats
Source: Plants (Basel). 2025 Jun 20;14(13):1893. doi: 10.3390/plants14131893 (PMC12251604; doi:10.3390/plants14131893)
Supplement: Supplementary file 1 [file plants-14-01893-s001.zip › plants-3651849-supplementary.pdf]

Supplimentary materials

Figure S1. Forest plot of parameter estimates (±95% confidence intervals) from a full factorial ANOVA model (PROC GLM) analysing seed germination percentage (gp) of *Salicornia brachiata* seeds. The model includes five categorical factors: temperature (t), perianth presence (p), seed type (s), chemical treatment (chem), and geographic location of the seed source (GL), including all main effects and their interactions (up to five-way). The y-axis lists the parameter names (main effects and interactions), while the x-axis shows the estimated effect sizes with 95% confidence intervals calculated as estimate ±1.96 × standard error. The vertical red line at zero represents no effect; estimates to the right indicate positive effects on germination, while those to the left indicate negative effects.

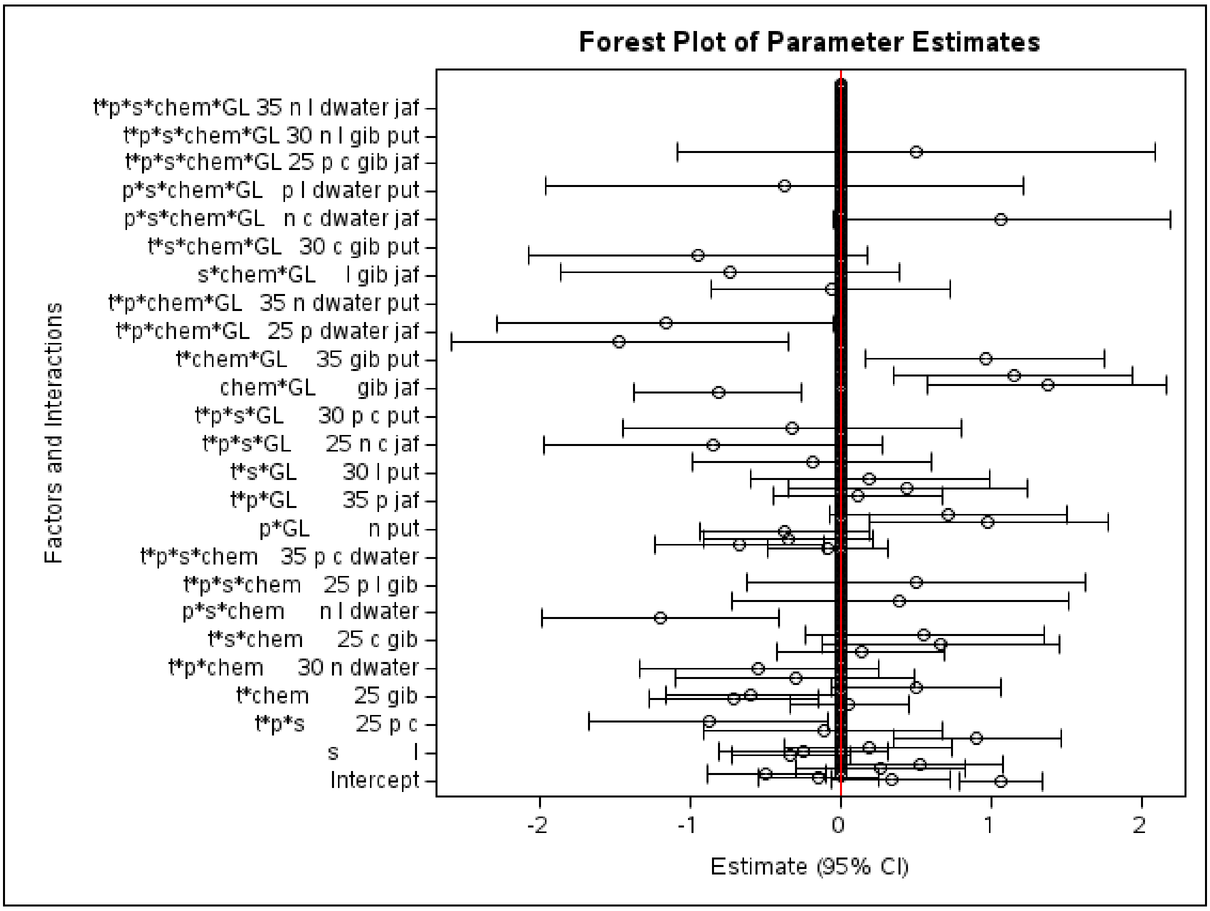

Table S1. Least square means (LS-means) and 95% confidence intervals for the four-way interactions among temperature (t), perianth presence (p), seed type (s), chemical treatment (chem), and geographic location of the seed source (GL) on seed germination percentage of *Salicornia brachiata* seeds. The table includes LS-means for the four-way interactions among: temperature × perianth presence × seed type ×

geographic location of the seed source; temperature × perianth presence × chemical treatment × geographic location of the seed source; and perianth presence × seed type × chemical treatment × geographic location of the seed source. All means were obtained according to a general linear model followed by Tukey's test.

Effect	t	p	s	GL	Estimate	StdErr	DF	tValue	P value	Alpha	Lower	Upper
t*p*s*GL	25	n	c	jaf	0.4667	0.10116	96	4.61	<.0001	0.05	0.2659	0.6675
t*p*s*GL	25	n	c	put	1.1417	0.10116	96	11.29	<.0001	0.05	0.9409	1.3425
t*p*s*GL	25	n	l	jaf	0.8050	0.10116	96	7.96	<.0001	0.05	0.6042	1.0058
t*p*s*GL	25	n	l	put	0.9367	0.10116	96	9.26	<.0001	0.05	0.7359	1.1375
t*p*s*GL	25	p	c	jaf	0.5433	0.10116	96	5.37	<.0001	0.05	0.3425	0.7441
t*p*s*GL	25	p	c	put	0.8817	0.10116	96	8.72	<.0001	0.05	0.6809	1.0825
t*p*s*GL	25	p	l	jaf	0.5800	0.10116	96	5.73	<.0001	0.05	0.3792	0.7808
t*p*s*GL	25	p	l	put	1.0667	0.10116	96	10.54	<.0001	0.05	0.8659	1.2675
t*p*s*GL	30	n	c	jaf	0.5483	0.10116	96	5.42	<.0001	0.05	0.3475	0.7491
t*p*s*GL	30	n	c	put	0.5133	0.10116	96	5.07	<.0001	0.05	0.3125	0.7141
t*p*s*GL	30	n	l	jaf	0.6150	0.10116	96	6.08	<.0001	0.05	0.4142	0.8158
t*p*s*GL	30	n	l	put	0.6433	0.10116	96	6.36	<.0001	0.05	0.4425	0.8441
t*p*s*GL	30	p	c	jaf	0.3567	0.10116	96	3.53	0.0006	0.05	0.1559	0.5575
t*p*s*GL	30	p	c	put	0.8333	0.10116	96	8.24	<.0001	0.05	0.6325	1.0341
t*p*s*GL	30	p	l	jaf	0.3717	0.10116	96	3.67	0.0004	0.05	0.1709	0.5725
t*p*s*GL	30	p	l	put	0.6400	0.10116	96	6.33	<.0001	0.05	0.4392	0.8408
t*p*s*GL	35	n	c	jaf	0.9200	0.10116	96	9.09	<.0001	0.05	0.7192	1.1208
t*p*s*GL	35	n	c	put	0.8850	0.10116	96	8.75	<.0001	0.05	0.6842	1.0858
t*p*s*GL	35	n	l	jaf	0.4600	0.10116	96	4.55	<.0001	0.05	0.2592	0.6608
t*p*s*GL	35	n	l	put	0.8450	0.10116	96	8.35	<.0001	0.05	0.6442	1.0458
t*p*s*GL	35	p	c	jaf	0.4050	0.10116	96	4.00	0.0001	0.05	0.2042	0.6058
t*p*s*GL	35	p	c	put	0.8233	0.10116	96	8.14	<.0001	0.05	0.6225	1.0241
t*p*s*GL	35	p	l	jaf	0.5950	0.10116	96	5.88	<.0001	0.05	0.3942	0.7958
t*p*s*GL	35	p	l	put	1.0900	0.10116	96	10.78	<.0001	0.05	0.8892	1.2908

Effect	p	s	chem	GL	Estimate	StdErr	DF	tValue	P value	Alpha	Lower	Upper
p*s*chem*GL	n	c	dwater	jaf	0.6744	0.08260	96	8.17	<.0001	0.05	0.5105	0.8384
p*s*chem*GL	n	c	dwater	put	0.5833	0.08260	96	7.06	<.0001	0.05	0.4194	0.7473
p*s*chem*GL	n	c	gib	jaf	0.6156	0.08260	96	7.45	<.0001	0.05	0.4516	0.7795
p*s*chem*GL	n	c	gib	put	1.1100	0.08260	96	13.44	<.0001	0.05	0.9460	1.2740
p*s*chem*GL	n	l	dwater	jaf	0.5967	0.08260	96	7.22	<.0001	0.05	0.4327	0.7606
p*s*chem*GL	n	l	dwater	put	0.7267	0.08260	96	8.80	.0001	0.05	0.5627	0.8906
p*s*chem*GL	n	l	gib	jaf	0.6567	0.08260	96	7.95	<.0001	0.05	0.4927	0.8206
p*s*chem*GL	n	l	gib	put	0.8900	0.08260	96	10.78	<.0001	0.05	0.7260	1.0540
p*s*chem*GL	p	c	dwater	jaf	0.2089	0.08260	96	2.53	0.0131	0.05	0.0449	0.3728
p*s*chem*GL	p	c	dwater	put	0.9233	0.08260	96	11.18	<.0001	0.05	0.7594	1.0873

p*s*chem*GL	p	c	gib	jaf	0.6611	0.08260	96	8.00	<.0001	0.05	0.4972	0.8251
p*s*chem*GL	p	c	gib	put	0.7689	0.08260	96	9.31	<.0001	0.05	0.6049	0.9328
p*s*chem*GL	p	l	dwater	jaf	0.3344	0.08260	96	4.05	0.0001	0.05	0.1705	0.4984
p*s*chem*GL	p	l	dwater	put	0.7400	0.08260	96	8.96	<.0001	0.05	0.5760	0.9040
p*s*chem*GL	p	l	gib	jaf	0.6967	0.08260	96	8.43	<.0001	0.05	0.5327	0.8606
p*s*chem*GL	p	l	gib	put	1.1244	0.08260	96	13.61	<.0001	0.05	0.9605	1.2884

Effect	t	p	chem	GL	Estimate	StdErr	DF	tValue	P value	Alpha	Lower	Upper
t*p*chem*GL	25	n	dwater	jaf	0.3983	0.10116	96	3.94	0.0002	0.05	0.1975	0.5991
t*p*chem*GL	25	n	dwater	put	0.8067	0.10116	96	7.97	<.0001	0.05	0.6059	1.0075
t*p*chem*GL	25	n	gib	jaf	0.8733	0.10116	96	8.63	<.0001	0.05	0.6725	1.0741
t*p*chem*GL	25	n	gib	put	1.2717	0.10116	96	12.57	<.0001	0.05	1.0709	1.4725
t*p*chem*GL	25	p	dwater	jaf	0.5067	0.10116	96	5.01	<.0001	0.05	0.3059	0.7075
t*p*chem*GL	25	p	dwater	put	0.8433	0.10116	96	8.34	<.0001	0.05	0.6425	1.0441
t*p*chem*GL	25	p	gib	jaf	0.6167	0.10116	96	6.10	<.0001	0.05	0.4159	0.8175
t*p*chem*GL	25	p	gib	put	1.1050	0.10116	96	10.92	<.0001	0.05	0.9042	1.3058
t*p*chem*GL	30	n	dwater	jaf	0.4850	0.10116	96	4.79	<.0001	0.05	0.2842	0.6858
t*p*chem*GL	30	n	dwater	put	0.2817	0.10116	96	2.78	0.0065	0.05	0.0809	0.4825
t*p*chem*GL	30	n	gib	jaf	0.6783	0.10116	96	6.71	<.0001	0.05	0.4775	0.8791
t*p*chem*GL	30	n	gib	put	0.8750	0.10116	96	8.65	<.0001	0.05	0.6742	1.0758
t*p*chem*GL	30	p	dwater	jaf	0.1733	0.10116	96	1.71	0.0899	0.05	0.0275	0.3741
t*p*chem*GL	30	p	dwater	put	0.6350	0.10116	96	6.28	<.0001	0.05	0.4342	0.8358
t*p*chem*GL	30	p	gib	jaf	0.5550	0.10116	96	5.49	<.0001	0.05	0.3542	0.7558
t*p*chem*GL	30	p	gib	put	0.8383	0.10116	96	8.29	<.0001	0.05	0.6375	1.0391
t*p*chem*GL	35	n	dwater	jaf	1.0233	0.10116	96	10.12	<.0001	0.05	0.8225	1.2241
t*p*chem*GL	35	n	dwater	put	0.8767	0.10116	96	8.67	<.0001	0.05	0.6759	1.0775
t*p*chem*GL	35	n	gib	jaf	0.3567	0.10116	96	3.53	0.0006	0.05	0.1559	0.5575
t*p*chem*GL	35	n	gib	put	0.8533	0.10116	96	8.44	<.0001	0.05	0.6525	1.0541
t*p*chem*GL	35	p	dwater	jaf	0.1350	0.10116	96	1.33	0.1852	0.05	0.0658	0.3358
t*p*chem*GL	35	p	dwater	put	1.0167	0.10116	96	10.05	<.0001	0.05	0.8159	1.2175
t*p*chem*GL	35	p	gib	jaf	0.8650	0.10116	96	8.55	<.0001	0.05	0.6642	1.0658
t*p*chem*GL	35	p	gib	put	0.8967	0.10116	96	8.86	<.0001	0.05	0.6959	1.0975
